# Characterization and Screening of Native *Scenedesmus* sp. Isolates Suitable for Biofuel Feedstock

**DOI:** 10.1371/journal.pone.0155321

**Published:** 2016-05-19

**Authors:** Rakesh Singh Gour, Aseem Chawla, Harvinder Singh, Rajinder Singh Chauhan, Anil Kant

**Affiliations:** Department of Biotechnology and Bioinformatics, Jaypee University of Information Technology, Solan, Himachal Pradesh, India; ICGEB, INDIA

## Abstract

In current study isolates of two native microalgae species were screened on the basis of growth kinetics and lipid accumulation potential. On the basis of data obtained on growth parameters and lipid accumulation, it is concluded that *Scenedesmus dimorphus* has better potential as biofuel feedstock. Two of the isolates of *Scenedesmus dimorphus* performed better than other isolates with respect to important growth parameters with lipid content of ~30% of dry biomass. *Scenedesmus dimorphus* was found to be more suitable as biodiesel feedstock candidate on the basis of cumulative occurrence of five important biodiesel fatty acids, relative occurrence of SFA (53.04%), MUFA (23.81%) and PUFA (19.69%), and more importantly that of oleic acid in its total lipids. The morphological observations using light and Scanning Electron Microscope and molecular characterization using amplified 18S rRNA gene sequences of microalgae species under study were also performed. Amplified 18S rRNA gene fragments of the microalgae species were sequenced, annotated at the NCBI website and phylogenetic analysis was done. We have published eight 18S rRNA gene sequences of microalgae species in NCBI GenBank.

## Introduction

Microalgae are among the most promising, renewable, non-food crop based alternative biofuel feedstocks due to several characteristics such as non-competition with food and feed crops, high oil content and growth rate [1]. Microalgae are microscopic photosynthetic organisms found in fresh, brackish and marine water. These organisms use solar energy to create biomass and accumulate triacylglycerides (TAGs), which can be converted into biodiesel via transesterification reaction [2, 3]. The mechanism of photosynthesis in microalgae is similar to higher plants but microalgae have higher photosynthesis efficiency, faster growth and can synthesize and accumulate larger quantities of lipids [2, 4, 5]. Algal biofuel production has not been commercialized yet due to high cost associated with production, inefficient harvesting and conversion of oil into biodiesel. Many technical challenges need to be addressed before microalgal biofuel becomes a commercial reality and one major challenge is to identify microalgal species/strains with high lipid productivity [6]. Selection of species/strains that are robust and display high growth and lipid accumulation rates is an important prerequisite for the success of microalgal biofuel in future. This may require exploration, identification and characterization of microalgae species and isolates of already known species from natural diversity. The hilly terrain of Himachal Pradesh (India) could be a potent source of algal biodiversity due to range of environmental conditions prevailing in the region. In current study we characterized and screened native isolates of *Scenedemus dimorphus* and *Scenedesmus quadricauda* collected and isolated previously [7] for growth rate, lipid content and productivity.

There are many reviews and reports available in the literature in which different species of microalgae have been discussed as potential biofuel feedstocks [8, 9] including *Scenedesmus* sp. as one of them. Griffiths and Harrison 2009 [8] reviewed information available in the literature on growth rates, lipid content and lipid productivities for 55 species of microalgae, as well as other taxa. The nutrient replete, lipid content of green algae ranged from 13% to 31% dw, with average of 23%, while average of *Scenedesmus dimorphus* was 26%. Many of the scientific reports claim very high accumulation of lipid content (>70%) in some microalgae species but under special nutrient starvation conditions that adversely affect the overall lipid productivity. The gain due to higher lipid content is counteracted by the lower productivities attained under nutrient shortage. This is one of major technical obstacle in realization of the algal biofuel and research efforts are required to explore and develop algal strains which defy this general principle. At the same time research is also needed to be done to devise and develop new methods to provide stress to algae culture which do not exert adverse effects on the growth. There is a need to isolate, screen, select, test and improve algal strains, for both higher oil content and overall productivity.

Traditionally taxonomic classification and subsequent identification often depended on morphological description of cell and colony features. Such observations being subjective sometimes result in double classification of the same organism and generates mistakes in the taxonomic assignation and identification [10–12]. Later in 1960s and 70s the concept of classification based on experimental studies of life cycles and architecture of flagellated cells was under taken [13, 14]. Mattox and Stewart 1984 [15] proposed a new classification based on the ultra-structure of the basal body in flagellated cells and cytokinesis during the mitosis. But most of such concepts are difficult to practice, particularly by non-taxonomists who are engaged in exploration of natural diversity of microalgae with an objective of bio-prospecting. The application of molecular markers as a tool in identification of microalgal species has been universally accepted [16–19] and there have been many studies on molecular characterization of microalgae. Molecular phylogenetic analyses, based on small subunit ribosomal DNA are largely congruent with ultra-structural knowledge, and seems easy to be practiced to identify the microalgae explored form natural diversity. This will be the gold standard for future taxonomic studies, provided the Gene bank sequence databases contain the characteristic sequences of large number of species found in different geographical regions. So here in we report amplification and sequencing of 18S rRNA gene fragment from three microalgae species collected from Himachal Pradesh in India. The sequences of all 18S rRNA gene fragments have been published in NCBI GenBank.

## Materials and Methods

### Microalgal samples

The freshwater isolates of microalgae species namely *Scenedesmus dimorphus* and *Scenedesmus quadricauda* and *Chlorella* sp. isolated previously from various locations of Himachal Pradesh, India (S1 Table) were used in this study [7]. Unialgal cultures of *Scenedesmus dimorphus* and *Scenedesmus quadricauda* and *Chlorella* sp. were isolated as per the method described in Gour et al 2014 [7] and cultures were regularly observed under light microscope for the purity of species. All the microalgae isolates were cultured in BG-11 medium [20] in subsequent experiments. The microalgae cultures were cultivated in a growth chamber in temperature controlled conditions at 24°C, illuminated with white fluorescent light of 3500 lux intensity for 16:8 hours of light and dark cycles.

### Growth kinetics and biomass estimation

The isolates of microalgal species under study were characterized for growth kinetics and biomass production potential to identify those which grew fast and accumulate higher biomass. 900 ml of BG-11 medium was inoculated with 100 ml inoculum of each isolate (10% v/v), adjusted to a cell density of 10–12 ×10^4^ cells ml^-1^. The cultures were aerated by air pump with 0.22 μm sterilized air filter to avoid settling and sticking to the surface of the flask. Optical density of microalgae cell culture suspensions was observed at 730 nm after every third day to monitor the stages of growth cycle. At the same time cell count (cells ml^-1^) was also recorded using a haemocytometer. When the stationary phase of growth reached, the cultures were centrifuged to harvest cell biomass, which was freeze dried to determine dry biomass content. Growth rate parameters of each isolate were calculated on the basis of cell count during exponential phase of growth and cell biomass. Following growth parameters were calculated from the observed data [21].

- Specific growth rate (μ): *μ = ln (Nt/N*_*o*_*)/Tt-T*_*o*_ where Nt is the number of cells at the end of log phase, N_o_ is the number of cells at the start of log phase, Tt is the final day of log phase and T_o_ is starting day of log phase.
- Doubling time: *Tt = 0*.*6931/μ*.
- Biomass productivity (Pdwt): as the dry biomass produced per day (g l^-1^ day^-1^).

### Estimation and quantification of lipid

Lipid accumulation estimation in microalgae biomass was done using modified Bligh and Dyer method [22] and indirect fluorescent spectrometric method [23]. Total lipids content (Lc) form former method was quantified in term of percent of dry biomass. Lipid productivity (Lp) was calculated according to the equation *Lp = Pdwt×Lc×1000/100* and expressed as milligrams per liter per day (mg L^-1^ day^-1^) [21].

For indirect estimation 15 ml of the culture from samples was centrifuged for 15 minutes at 7000 rpm, supernatant was discarded and the pallet was stored at –80°C. 2 ml of chloroform and 4 ml of methanol were added to the vials, which were then incubated for 45 minutes in a water bath at 45°C with intermittent mixing on vortex shaker for extraction of lipids. This solution was not disturbed for some time so as to separate it in two phases. 1.5 ml of light fraction (lipids in chloroform) was taken for staining with 5μL of 10^−4^ mol L^–1^ Nile Red (Sigma) dissolved in acetone according to Elsey et al [24]. The fluorescence of the lipid samples was recorded by exciting the samples at 486 nm and the emission was confirmed at 570 nm using a Jasco FP 750 spectrofluorometer. Emission intensities were recorded over a period of 300 seconds.

### Fatty acid profile of total lipids of microalgae species

Fatty acid profile analysis of species wise pooled total lipids isolated from different isolates of a microalgae was carried out using gas chromatography mass spectroscopy (GC-MS) at CIL/SAIF at Panjab University Chandigarh, India. Fatty acid methyl esters were prepared using following procedure. 30 mg of total lipid dissolved in 1 ml of methanol was mixed with 1 ml of 12% solution of KOH prepared in methanol. To this solution equal volume of 5% HCL in methanol was added and heated at 75°C for 15 min. This solution was allowed to cool and 1 ml of distilled water was added and shaken. Upper organic layer containing fatty acid methyl esters was carefully transferred to a new clean vial. GC-MS analysis of FAMEs was performed using diethylene glycol succinate capillary column (30m × 0.25 × 0.25μm). 100μl of methyl ester sample solution was injected for each analysis. Helium was used as a carrier gas. The injector temperature was 180°C and detector temperature was 230°C which was increased to 300°C at a temperature gradient of 15°C/min. Identification of fatty acid methyl ester was done by comparing the mass spectra with NIST database library [25].

### Statistical analyses

Data were expressed as mean of three independent parallel experiments. ANOVA was used to assess the differences amongst the treatments. Standard error of means values were calculated at <0.05 level of significance. The superscripts in tables represent the positions based on Tukey HSD Multiple Range test and different alphabets denote significantly different values as analyzed using SPSS 17. Five percent error is depicted in data presented as bar diagrams.

### Cell and Colony Morphology

Morphological observations on cells and colonies of microalgae species were carried out using an OLYMPUS 100× light microscope (LM) and digital images were captured. Scanning Electron micrograph (SEM) observations of representative samples of microalgae were also carried out to visualize three dimensional shape and size of cells. For SEM, 1 ml cultures were harvested [26] and fixed with glutaric dialdehyde for 2 to 4 hrs. The cells were coated with gold and examined under the SEM microscope (Hitachi) (CIL, NIPER, Mohali India).

### DNA isolation, 18S region amplification

Molecular phylogenetic analysis of three microalgae species was done for future reference and identity of the species. One isolate from each species viz *Sd12*, *Sq2*, *Chl1*, from *S*. *dimorphus*, *S*. *quadricauda* and *Chlorella* sp. were included randomly in this analysis. 50 ml of microalgae cultures were centrifuged at 7000 rpm for 10 minutes and pellet crushed in cold mortar and pestle to a fine powder by using liquid nitrogen. Subsequently microalgal DNA was isolated by using the Pure Link Plant Total DNA Purification Kit (Invitrogen), following the manufacturer's instruction. The quality of DNA was checked by visualization of genomic DNA after gel electrophoresis in 0.8% agarose in gel doc system. The 18S rRNA gene fragment of each microalgae species was amplified using green microalgae specific primer pairs previously described in literature (Table 1) [27, 28]. PCR was performed in 15μl reaction containing 0.6μM of each primer, 5mM dNTPs, 30 ng templates DNA, 1× PCR buffer, and 1.0 U of Taq DNA polymerase. The PCR program included an initial denaturation of 4 minutes at 94°C followed by 35 cycles of denaturation at 94°C for 1 minute, primer annealing at 52.5°C for 55 seconds, primer extension at 72°C for 1 minute and final extension at 72°C for 5 minutes. The PCR products were separated by electrophoresis, using 1.5% agarose and visualized in Bio-Rad Gel doc system. The amplified PCR products were directly sequenced (Xcelris Labs Ltd, Ahmadabad 380054 India) via Sanger’s chain termination method using same primers.

**Table 1:** PCR primers pairs used for the amplification of 18S rRNA gene fragments from microalgae under study and accession numbers amplified sequences published in NCBI Gene Bank.

S. No.	Species	Primer ID	Accession No.	Size
1	*Scenedesmus quadricauda*	F- P73, R- P47	KC790428	270 bp
2	*Scenedesmus quadricauda*	F-CV3, R-CV4	KC790429	748 bp
3	*Scenedesmus dimorphus*	F- P73, R- P47	KC790430	273 bp
4	*Scenedesmus dimorphus*	F-CV3, R-CV4	KC790431	747 bp
5	*Chlorella* sp.	F- P73, R- P47	KC790432	276 bp
6	*Chlorella* sp.	F-Chloro, R-Chloro	KC790433	396 bp
7	*Chlorella* sp.	F-CV1, R-CV2	KC790434	1000 bp
8	*Chlorella* sp.	F-CV3, R-CV4	KC790435	746 bp

### Sequence annotation and phylogenic analysis

The longest 18S rRNA amplified DNA fragment (i.e. fragment amplified by CV3-CV4) of the three microalgae under study was annotated with genus specific sequences from NCBI GenBank database using the basic local alignment search tool (BLAST). Ten genus specific sequences showing the highest similarity scores with each query sequence were retrieved from NCBI GenBank database. Multiple sequence alignment was carried out among retrieved and query sequences using the CLUSTAL-X program and phylogenetic tree was constructed using the software Mega 5.0.2 [29]. The unweighted pair group arithmetic mean (UPGMA) method was used for phylogenetic tree reconstruction and evolutionary distances were computed using the p-distance method. Bootstrap values based on the analysis of 500 bootstrap replicates were calculated to estimate the degree of confidence assigned to the nodes in the phylogenetic trees [30]. The tree was drawn to scale, with branch lengths in the same units as those of the evolutionary distance [31].

## Results and Discussion

### Characterization of microalgae species for growth and lipid content

**Fig 1:**
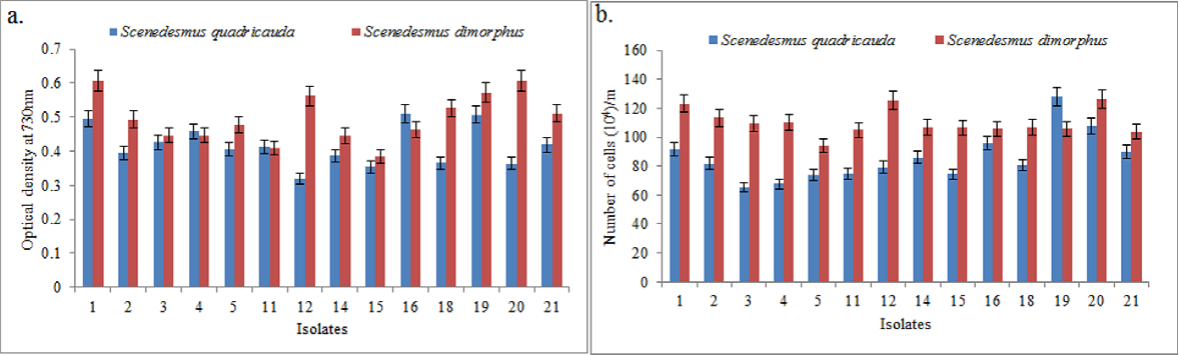
**Growth response of microalgal species isolates observed on 18^th^ day (a) measured as OD at 730 nm and (b) measured as cell density per ml.** The values are mean of three replicates and error bar shown are 5% of the mean value.

**Fig 2:**
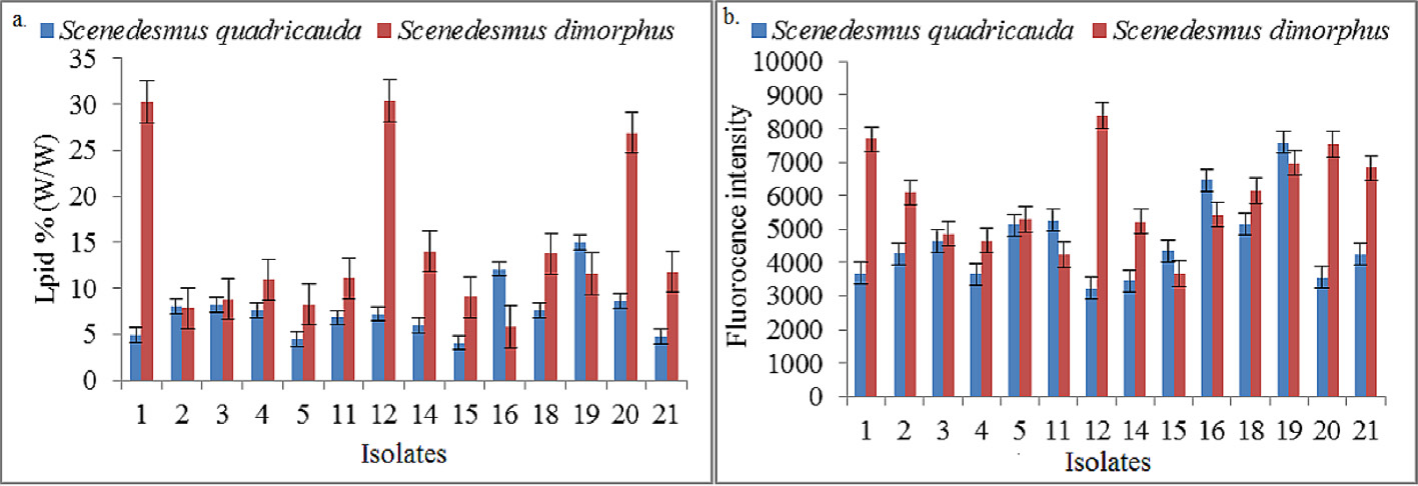
Accumulation of total lipid in isolates of microalgal sp. over 18^th^ day of cultivation, (a) Direct gravimetric estimation using modified Bligh & dyer method; Total lipid expressed as percentage (w/w) in dried biomass. (b) Quantified by the Nile red fluorescence method. Data were the mean values and standard error of three replicates.

The growth response of isolates of *Scenedesmus dimorphus* and *Scenedesmus quadricauda* measured as optical density at 730 nm and cell density on 18^th^ day of culture is presented in Fig 1A and 1B. The algae species under study had a growth cycle of 16 days to reach stationary phase [7]. A fair range of variability was observed among different isolates of both the species with respect to optical density and cell count. The isolates *Sd1*, *Sd12 & Sd20* of *Scenedesmus dimorphus* and *Sq16*, *Sq19* of *Scenedesmus quadricauda* displayed better performance compared to others within the same species. All the isolates of both the species were further characterized for the growth rate, biomass and lipid accumulation potential. Biomass productivity and lipid content are two most studied parameters in search of the prominent isolates for large-scale cultivation of microalgae for biofuel production [8]. The data pertaining to growth kinetics and lipid accumulation parameters of *Scenedesmus dimorphus* and *Scenedesmus quadricauda* is shown in Tables 2 and 3 respectively. The average specific growth rate of the *Scenedesmus dimorphus* isolates ranged from 0.113 to 0.148; isolate *Sd1*, followed by *Sd20* displayed the highest. Both these isolates performed better than others with respect to important growth parameters viz. biomass productivity, lipid content and lipid productivity as well (Table 2). The lipid content of isolate *Sd12* and *Sd1* was 30.39% and 30.23% respectively measured as percentage of dry biomass (Table 2, Fig 2A). The amount of total lipids in these isolate was also estimated by indirect fluorescent spectrometric method and results are presented in Fig 2B. Here also highest fluorescence was recorded for isolate *Sd12*, followed by *Sd1*. The lipid content of ~30% of dry biomass is moderately high under the nutrient replete conditions. According to Griffiths and Harrison 2009 [8] average of lipid content of *Scenedesmus dimorphus* reported in the literature was 26%. Higher growth rate, lipid content and productivity are generally negatively correlated that is why exploration is necessary to identify such isolates which deviate from this and have combination of good traits. Velichkova et al (2013) [32] conducted a study on *Scenedesmus dimorphus* and found that lipid content in the examined strain was 21.6% in BBM and 18.5% in 3N-BBM medium. Jena et al (2012) [33] screened three brackish water microalgal strains (*Chlorococcum* sp., *Chlorella* sp. and *Scenedesmus* sp.) of Odisha coast for the suitability for biodiesel production. They found *Scenedesmus* sp. to be the best one for high lipid productivity biomass yield. Ren et al (2013) [34] reported selection of a novel green microalga *Scenedesmus* sp. strain R-16 with high total lipid content. Compared to autotrophic situation, the strain R-16 grew well heterotrophically without light and the accumulated total lipid content and biomass reached 43.4% and 3.46 g L^-1^, respectively. Under nitrogen starvation the total lipid content was as high as 52.6%. From the data obtained and literarature reports so far on the *Scenedesmus* species, it can be concluded that isolates *Sd1* and *Sd12* of *Scenedesmus dimorphus* clearly diverge from the rest and thus can be considered for the further research on standardization of cultivation methods and strain improvement.

**Table 2:** Growth kinetics, biomass, biomass productivity, lipid content, and lipid productivity of *Scenedesmus dimorphus* isolates.

Isolates	Specific growth rate (μ)***	Doubling Time(Tt) **	Biomassg l^-1^***	Biomass productivitygl^-1^day^-1^***	Lipids content (Lc) %***	Lipid productivity mg l^-1^day^-1^^ns^
*Sd1*	0.148^e^	4.683^ab^	0.255^abcd^	0.0134 ^abcd^	30.23^b^	4.051 ^a^
*Sd2*	0.115^de^	6.026^ab^	0.211^ab^	0.0111 ^ab^	7.86 ^a^	0.872 ^a^
*Sd3*	0.135^bcde^	5.13^ab^	0.299^de^	0.0157 ^cd^	8.83 ^a^	1.386 ^a^
*Sd4*	0.127^abcd^	5.44^b^	0.277 ^bcde^	0.0145 ^bcd^	10.95 ^a^	1.588 ^a^
*Sd5*	0.113^a^	6.133^b^	0.244^abcd^	0.0116 ^abc^	8.22 ^a^	0.953 ^a^
*Sd11*	0.131^abcd^^e^	5.29^ab^	0.244^abcd^	0.0128 ^abcd^	11.08 ^a^	1.418 ^a^
*Sd12*	0.126^abcd^	5.50^a^	0.299^de^	0.0140 ^bcd^	30.39^b^	4.255 ^a^
*Sd14*	0.140^cde^	4.95^ab^	0.188^a^	0.0098^a^	14.01 ^a^	1.373 ^a^
*Sd15*	0.135^bcde^	5.13^ab^	0.222^abc^	0.0116 ^abc^	9.05 ^a^	1.049 ^a^
*Sd16*	0.120^ab^	5.77^b^	0.288^cde^	0.0169 ^de^	5.83^a^	0.985 ^a^
*Sd18*	0.128^abcd^	5.41^b^	0.244^abcd^	0.0128 ^abcd^	13.76 ^a^	1.761 ^a^
*Sd19*	0.122^abc^	5.68^b^	0.344^e^	0.0200 ^e^	11.58 ^a^	2.316 ^a^
*Sd20*	0.141^cde^	4.91^ab^	0.211^ab^	0.0111 ^ab^	26.91^b^	2.987 ^a^
*Sd21*	0.120^ab^	5.77^b^	0.222^abc^	0.0105^ab^	11.77 ^a^	1.236 ^a^

*Significant at *α* = 5%

**Significant at *α* = 1%

***Significant at *α* = 0.1%

^**ns**^Non-significant,level of significance.

a, b, c, d, e, f, g means in the column with same superscript letter are not significantly different (α = 0.05) as measured by 2 sided Tukey’s–post-hoc range test between isolates.

**Table 3:** Growth kinetics, biomass, biomass productivity, lipid content, and lipid productivity of *Scenedesmus quadricauda* isolates.

Isolates	Specific growth rate (μ)***	Doubling Time(Tt) **	Biomassg l^-1^***	Biomass productivityg l^-1^day^-1^***	Lipids content(Lc) % ***	Lipid productivitymg l^-1^day^-1^***
*Sq1*	0.128 ^bcd^	5.414 ^bcde^	0.210 ^ab^	0.011 ^abc^	4.92 ^a^	0.541 ^ab^
*Sq2*	0.112 ^abc^	6.188 ^efg^	0.211 ^ab^	0.011 ^abc^	8.09 ^ab^	0.889 ^abc^
*Sq3*	0.125 ^bcd^	5.544 ^bcdef^	0.244 ^abc^	0.014 ^cde^	8.22 ^ab^	1.151 ^bc^
*Sq4*	0.110 ^ab^	6.300 ^fg^	0.222 ^abc^	0.012 ^abcd^	7.62 ^ab^	0.914 ^abc^
*Sq5*	0.122 ^abcd^	5.681 ^bcdef^	0.222 ^abc^	0.013 ^bcde^	5.52 ^a^	0.718 ^ab^
*Sq11*	0.106 ^a^	6.538 ^g^	0.244 ^abc^	0.013 ^bcde^	6.79 ^ab^	0.882 ^abc^
*Sq12*	0.139 ^de^	4.986 ^ab^	0.277 ^bc^	0.018 ^f^	7.22 ^ab^	1.299 ^cd^
*Sq14*	0.113 ^abc^	6.133 ^defg^	0.222 ^abc^	0.011 ^abc^	5.95 ^a^	0.615 ^abc^
*Sq15*	0.119 ^abc^	5.824 ^cdefg^	0.244 ^abc^	0.014 ^bcde^	4.11 ^a^	0.575 ^ab^
*Sq16*	0.128 ^cd^	5.414 ^abc^	0.277 ^bc^	0.016 ^def^	12.10 ^bc^	1.936 ^de^
*Sq18*	0.105^a^	6.600 ^g^	0.211 ^abc^	0.0111 ^abc^	7.62 ^ab^	0.846 ^abc^
*Sq19*	0.150^e^	4.620 ^a^	0.288 ^c^	0.016 ^ef^	14.99 ^c^	2.398 ^e^
*Sq20*	0.130 ^cd^	5.331 ^abcd^	0.188 ^a^	0.0098 ^a^	8.67 ^ab^	0.849 ^abc^
*Sq21*	0.119 ^abc^	5.824 ^cdefg^	0.211 ^ab^	0.011 ^ab^	4.78 ^a^	0.525 ^a^

*Significant at *α* = 5%

**Significant at *α* = 1%

***Significant at *α* = 0.1%, level of significance.

a, b, c, d, e, f, g means in the column with same superscript letter are not significantly different (α = 0.05) as measured by 2 sided Tukey’s–post-hoc range test between isolates.

On the other hand similar variation were observed in most of the growth kinetic parameters of *Scenedesmus quadricauda*, and isolates *Sq12*, *Sq16* and *Sq19* performed better than other isolates of this species. Highest lipid content of 14.99% and fluorescence intensity was recorded in case of isolate *Sq19* (Table 3, Fig 2A and 2B). The lipid content of best performing isolate of *Scenedesmus quadricauda* was low compared to those of *Scenedesmus dimorphus*. So it can be concluded that *Scenedesmus dimorphus* isolates has better potential as biofuel feedstock. However best and worst performing isolates of both the species are being maintained, persevered and will be submitted to national algal repositories as these can serve as valuable characterized germplasm for future research viz. comparative expression of genes so as to decipher gene involved to confer their characteristic traits.

### Fatty acid profiling of potential *Scenedesmus* species isolates

**Fig 3:**
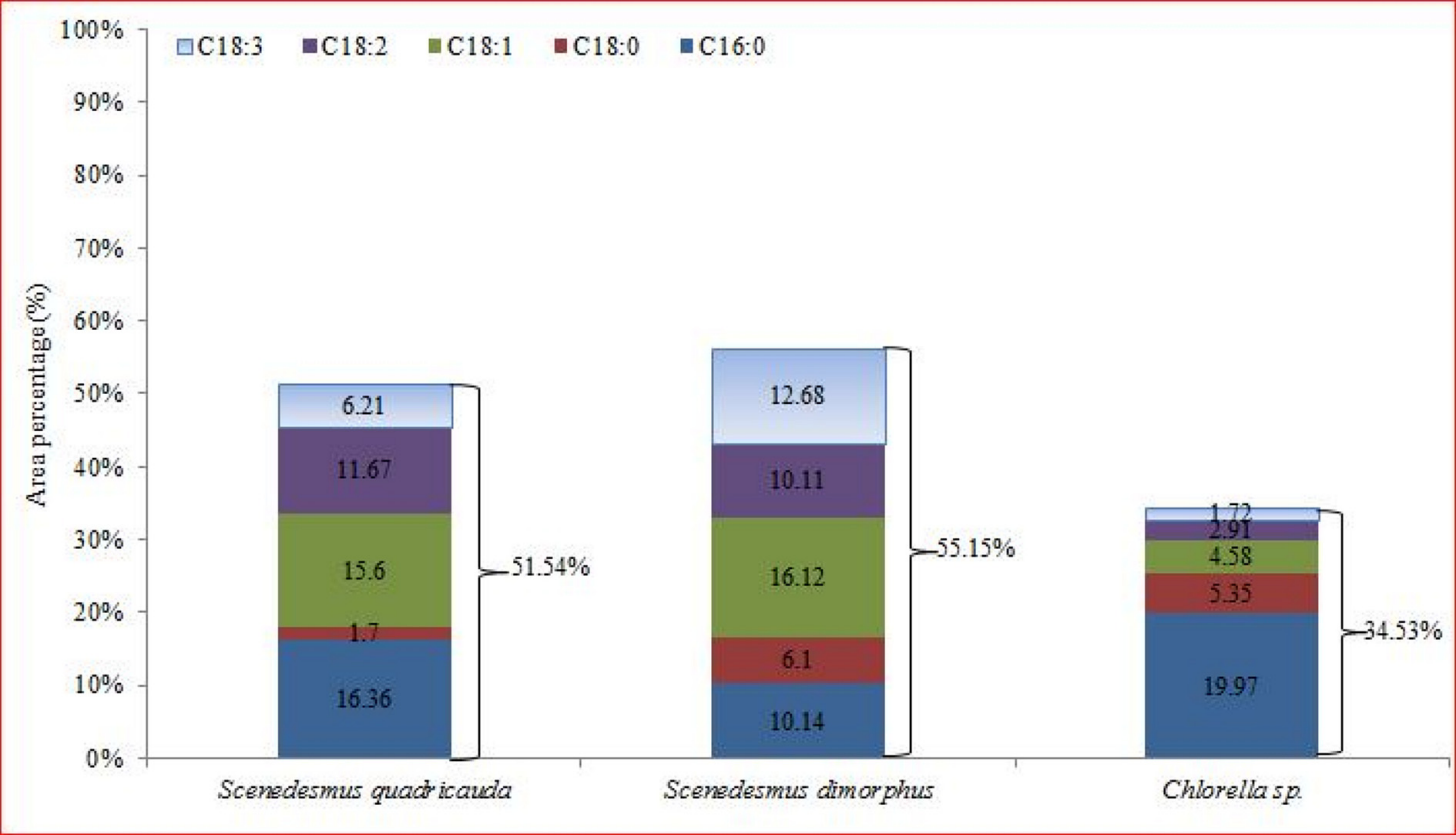
Cumulative occurrence of five important fatty acids commonly found in biodiesel in total lipids in microalgal species.

The list of fatty acids and their relative proportion in term of area represented by the specific peak in GC-MS profile of total lipids of three microalgae species is given in S2, S3 and S4 Tables Twenty fatty acid methyl esters (FAMEs) of varying carbon length were detected in the lipids of microalgae under study. Five major fatty acids in *Scenedesmus dimorphus* were oleic acid (16.12%), linolenic acid (12.68%), palmitic acid (10.14%), linoleic (10.11%) and 2-methy tetracosane (6.83%). *Scenedesmus quadricauda* lipids contained palmitic acid (16.36%), oleic acid (15.60%), linoleic acid (11.67%). 3, 7, 11- trimethyl-2, 4-dodecadiene (9.67%) and linolenic acid (6.21%). Similarly palmitic acid (19.97%), hexadecane, 2, 6, 11, 15- tetra methyl (C20:2), (9.85%), heptadecane-2, 6, 10, 15 tetramethyl (8.15%), Heneicosane, 11 (1-ethylpropyl) (6.27) and Phytol (5.95%) were most abundant fatty acids in the total lipids of *Chlorella* sp. Many of the parameters of biodiesel such as cetane number, kinematic viscosity, oxidative stability and cold flow properties depend on the fatty acid profile of biodiesel oil [35]. Very short chain fatty acids, very long chain fatty acids and highly unsaturated fatty acids are undesirable in biodiesel feedstock. The common fatty methyl esters found in biodiesel extracted from plants like soyabean, canola and palm include palmitic acid, stearic acid, oleic acid, linoleic acid and linolenic acid [36]. The cumulative occurrence of these five important fatty acids in three microalgae species is depicted in Fig 3. It was found that *Scenedesmus dimorphus* contained highest amount of desirable fatty acids (55.15%) followed by *Scenedesmus quadricauda* (51.54%) among the microalgae under study. The relative occurrence of saturated fatty acid (SFA), mono unsaturated fatty acid (MUFA) and poly unsaturated fatty acid (PUFA) is also depicted in S2, S3 and S4 Tables. SFA (*S*. *dimorphus* 38.03, *S*. *quadricauda* 53.04%) followed by PUFA (*S*. *dimorphus* 39.03, *S*. *quadricauda* 19.69%) occurred in higher proportion in both of *Scenedesmus* species, whereas MUFA was observed highest in *Chlorella* sp. (48.2%). The relative degree of unsaturation and saturation of fatty acids in biodiesel feedstock influence the biodiesel properties [37]. Cetane number is one of the prime indicators of biodiesel quality and it should be greater than 51 according to most of standards of biodiesel quality such as ASTMD 6751 and UNE-E14214. Cetane number decrease with unsaturation of fatty acids in biodiesel feedstocks [37, 38]. Similarly other properties such as oxidative stability, heat of combustion, viscosity of biodiesel increase with chain length and decrease with level of unsaturation [37]. The level of unsaturation of *Scenedesmus* species oils was comparable or slightly better to that of soybean oil (SFA 15.3%, MUFA 25.6%, PUFA 59.1%) [37]), which is one of leading biodiesel feedstock used worldwide. A significant higher percentage of oleic acid was observed in *Scenedesmus dimorphus* (16.12%) and *Scenedesmus quadricauda* (15.6%) compared to *Chlorella* sp. (4.58%) (Fig 3). Rodolfi et al. 2009 [39] also reported highest oleic acid content in *Scenedesmus* sp. among microalgal species they investigated. It has been reported by many workers [35, 40, 41] that oleic acid methyl esters in biodiesel improve fuel properties of biodiesel. Jena et al (2012) [33] found *Scenedesmus* sp. to possesses the most adequate fatty acid profile among the three brackish water microalgal strains screened (*Chlorococcum* sp., *Chlorella* sp. and *Scenedesmus* sp.) isolated from Odisha coast of India. Their study suggested that *Scenedesmus* sp. is appropriate for bio-diesel production for its high lipid content and selected this strain for higher scale studies. Similarliy Prabakaran and Ravindran (2012) [42] found *Scenedesmus* sp. to conatain highest amount of oleic acid (11.77 mg g^–1^dry wt) among different microalgal cultures (*Chlorella*, *Haematococcus*, *Ulothrix*, *Chlorococcum*, *Scenedesmus*, *Rivularia* and *Scytonema*) isolated from six different water bodies from Gandhigram, Tamil Nadu. Their results suggested that *Scenedesmus* sp. is useful for producing biodiesel, based on its high lipid and oleic acid contents. Thus on the basis of cumulative occurrence of five important biodiesel fatty acids, relative occurrence of SFA, MUFA and PUFA and more importantly oleic acid, and literature reports it is concluded that *Scenedesmus dimorphus* is one the of suitable candidate as biodiesel feedstock.

### Comparison of Growth and lipid production potential with relevant studies

A number of microalgae species have been investigated for growth and lipid production potential previously and as such there is no clear cut model species which can be included in experimental set up as standard species to make comparisons. In such a scenario it was thought of comparing the results with similar type of studies from the literature. Such comparison has been also made by Ren et al 2013 and Rodolfi et al., 2009 [8, 34, 39]. The data pertaining to growth, lipid productivity and lipid profile analysis of different algal species in somewhat similar type of studies are presented in the Table 4.

**Table 4:** Comparison of biomass productivity, lipid content and productivity and fatty acid composition of some microalgae reported in similar studies with *S*. *dimorphus* isolates *Sd12* and *Sd1*.

Microalgae species	Biomass productivity	Total lipid content	Lipid productivity	Growth condition	Relative % of fatty acids			References
	(g/L/day)	(%)	(mg/L/day)		MUFA	PUFA	SFA	
*Sd12*	0.0140	30.39	4.255	Autotrophic, replete without CO_2_	22.88	39.03	38.09	This study
*Sd1*	0.0134	30.23	4.051	Autotrophic, replete without CO_2_	NA	NA	NA	This study
*Chl16* sp.	0.015	27.60	4.140	Autotrophic, replete without CO_2_	48.24	22.05	24.04	[7]
*Chlamydomonas* sp.	0.30	15.02	36.17	Autotrophic, replete with CO_2_	14.63	06.76	78.61	[44]
*UTEX 2219–4*	NA	18.48	NA	Autotrophic, replete without CO_2_	NA	NA	NA	[49]
*Scenedesmus obliquus*	0.16	16.73	26.77	Autotrophic, replete with CO_2_	21.71	07.46	70.83	[44]
*Desmodesmus brasiliensis*	0.13	17.99	23.39	Autotrophic, replete with CO_2_	44.08	21.38	34.54	[44]
*Scenedesmus quadricauda*	0.19	18.4	35.1	Autotrophic, replete with CO_2_	NA	NA	NA	[39]
*Scenedesmus* sp. *DM*	0.26	21.1	53.9	Autotrophic, replete with CO_2_	NA	NA	NA	[39]
*Scenedesmus F&M-19*	0.21	19.6	40.8	Autotrophic, replete with CO_2_	NA	NA	NA	[39]
*Chlorococcum* sp.	NA	12.5	12.44	Autotrophic, replete with CO_2_	NA	79.7	20.3	[33]
*Chlorella* sp.	NA	15.5	16.11	Autotrophic, replete with CO_2_	NA	66.0	34.0	[33]
*Scenedesmus* sp.	NA	24.0	24.66	Autotrophic, replete with CO_2_	NA	63.5	36.5	[33]
*Chlorella sorokiniana*	NA	NA	NA	Autotrophic, replete	8.0	60.2	31.8	[50]

The specific growth rate and lipid productivity figures in most of studies are higher from the our isolates. It may be attributed to the fact that in most of these studies, algal culture were flushed with carbon dioxide which may be responsible for higher growth rates and biomass. It may be noted that this is a preliminary study in which primary focus was to screen the best performing isolates of the indigenous microalge species. The isolates *Sd1* and *Sd12* can be considered for the further research and productivity can be improved on standardization of cultivation methods and strain improvement.

The lipid content of the isolates *Sd12* and *Sd1* (~30%) was found to be higher as compared that of in all of such studies (Table 4) and that too without any stress and nutrient replete conditions. The lipid content of numerous isolates of microalgae reported previously ranged from 5–45 (% dwt) depending on the species and environmental, nutrients and stress conditions [8]. It is also important to mention here that lipid production potential of the microalgae isolates was evaluated under normal growth conditions, without any added energy source, CO_2_ supply and stress in order to reveal their true genetic potential to produce and accumulate lipids. Very high lipid productivities reported so for are achieved in heterotrophic and or mixotrophic culture conditions, where in culture supplied with some carbon source. This adversely affect the positive energy balance of lipid production and may not be practical and economical at commercial scale. For example Ren et al 2013 [34] achieved lipid productivity of 250.27 mg^-1^day^-1^ with *Scenedesmus dimorphus* R-16 strain, using culture medium supplemented with 10 mg l^-1^ of glucose. The *Scenedesmus dimorphus* was found to be suitable on the basis of fatty acid profile and composition as discussed above. Many workers have reported *Scenedesmus* sp. as potential source of algal oil production in terms of productivity and oil quality [42–47]. We have isolated and characterized the better performing native isolates of the *Scenedesmus dimorphus*, the productivity of which can be further improved by optimizing cultural methods and genetic interventions.

### Morphological and molecular characterization

**Fig 4:**
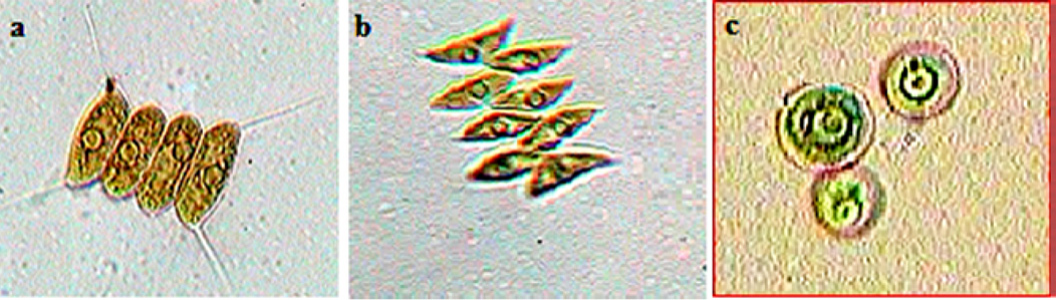
Cells and colonies of microalgae species as observed under light microscope (100X), (a) *Scenedesmus quadricauda*, (b) *Scenedesmus dimorphus* and (c) *Chlorella* sp.

**Fig 5:**
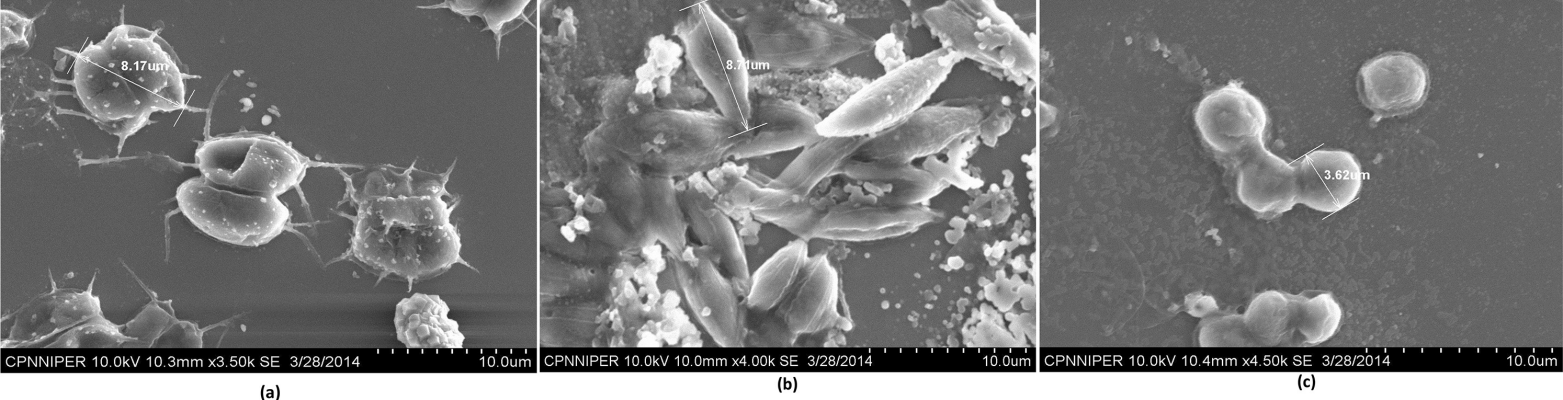
**Scanning electron micrographs of *Scenedesmus quadricauda* (a), (b) *Scenedesmus dimorphus* and (c) *Chlorella* sp.** a: Magnification 3500×; b: Magnification 4000×; c: Magnification 4500×.

The cell morphology of three of microalgae species as observed under the LM and SEM is depicted in Figs 4 and 5. Three dimensional shapes of *Chlorella* sp. (isolated from the same samples) were spherical where as that of *Scenedesmus quadricauda* and *Scenedesmus dimorphus* were near to spherical cuboidal and spherical lanceolate respectively. *Scenedesmus quadricauda* and *Scenedesmus dimorphus* formed typical colonies consisting of four or eight cells although two cell formation and single cells were also observed. The cells of *Chlorella* sp.were mostly free living sometimes aggregating into numerous cells. The length of longer axis of *Scenedesmus quadricauda* and *Scenedesmus dimorphus* ranged from 6.21 to 8.3 μm and 8.28 to 8.74 μm respectively whereas diameter of *Chlorella* sp. cells ranged between 3.47–3.62μm.

**Fig 6:**
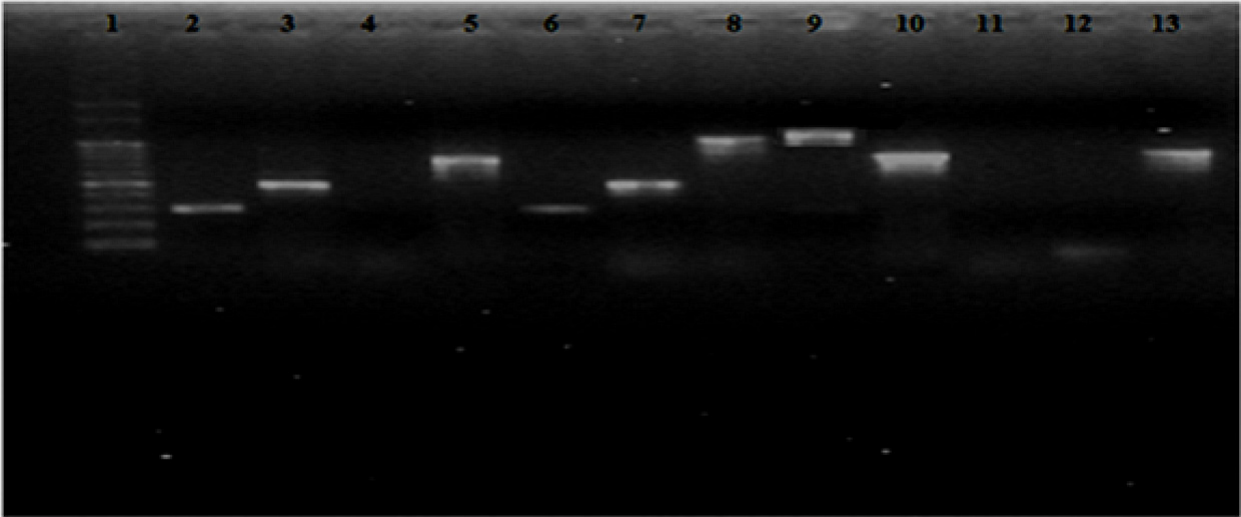
Amplified 18S rRNA gene fragments using P73-P47, Chloro F &R, CV1-CV2, CV3-CV4 primer pairs from genomic DNA of *Scenedesmus quadricauda* (lane 2,3,4, 5), *Chlorella* sp. (Lane 6,7,8,9) *Scenedesmus dimorphus* (Lane 10,11,12,13) Lane 1 is 100 bp ladder.

DNA was extracted in sufficient amount and the quality suitable for PCR amplification from three microalgae using Pure Link Plant Total DNA Purification Kit (Invitrogen, India). The 18S rRNA specific primer pairs P73-P47 and CV3- CV4 amplified 18S rRNA gene fragments from all three microalgae. Primer pair CV1- CV2 did not amplify the same from *Scenedesmus dimorphus* while Chloro F-R amplified from *Chlorella* sp. only. The amplified 18S rRNA gene fragments ranged between 300–1000 bp in size Fig 6. The sequences of these fragments have been published in NCBI Gene Bank database under accession numbers KC790428 to KC790435 (Table 1). The longest amplified 18S rRNA gene sequences amplified by CV3-CV4, showed 92–98% sequence similarity with 94–98% query coverage with partial 18S rRNA gene sequence from different species of same genus (S5 Table). NCBI database did not contain the target species specific sequences so microalgae under study could not be identified up to species level on the basis of 18S rRNA gene sequence similarities, as more representative sequences of microalgae described up to species level are needed in public databases to make identification straight forward. We published eight sequences in NCBI data base (Table 1) and such sequence submissions are required to enrich gene databases.

**Fig 7:**
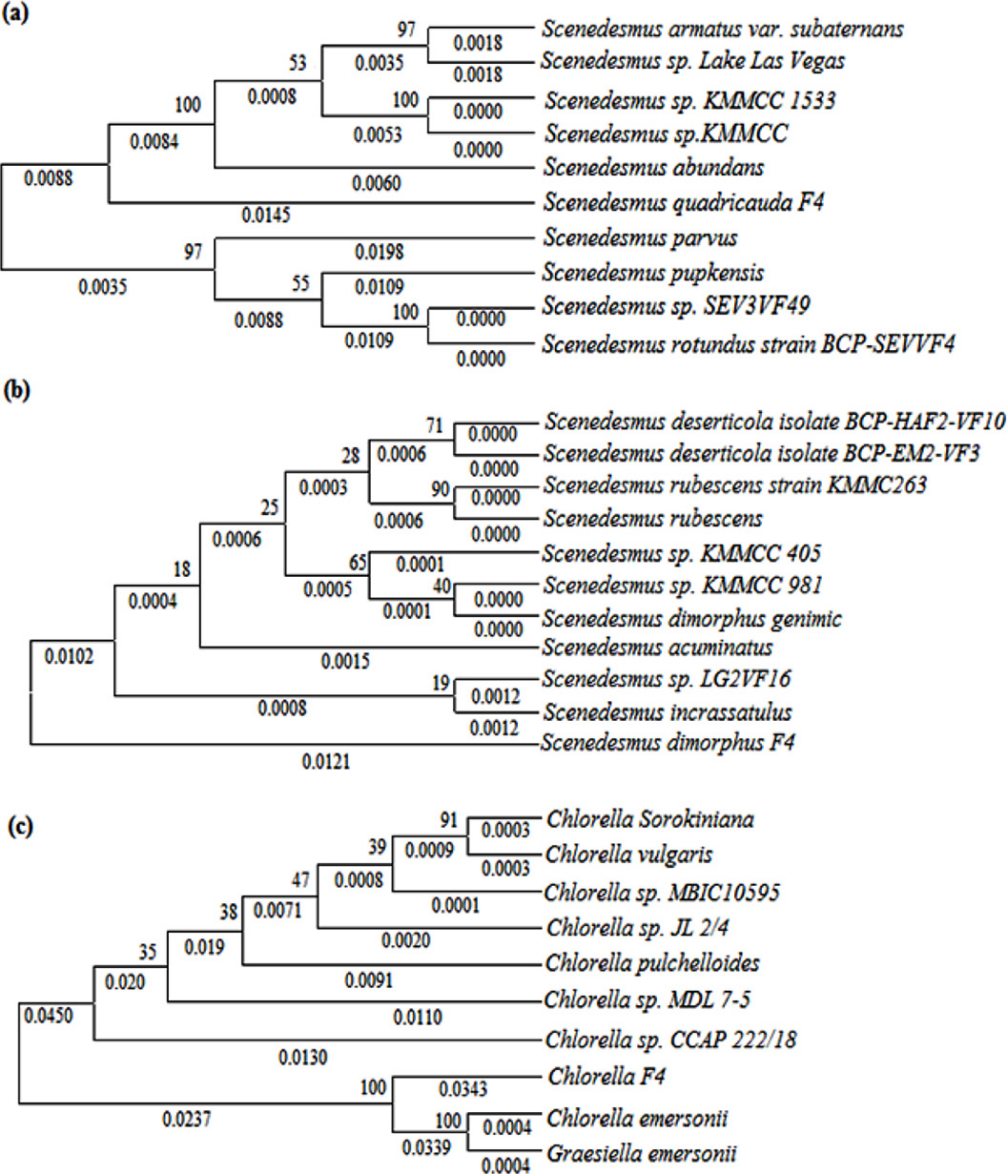
**Phylogenetic trees of microalgae (a) *Scenedesmus quadricauda*, (b) *Scenedesmus dimorphus* and (c) *Chlorella* sp. inferred from alignment of their 18S rRNA gene fragment sequences and ten most similar sequences of same genus retrieved from NCBI database.** The query sequence of respective species of this study designated as name of the specie followed by F4.

The ITS1 and ITS2 sequences are significant alternative markers for studying the phylogenetic relationship within the *Scenedesmaceae* [48]. The phylogenetic tree of the microalgae viz; *Scenedesmus quadricauda*, *Scenedesmus quadricauda* and *Chlorella* sp. constructed using the longest 18S rRNA query sequence and ten sequences from database showing the highest scores and maximum query coverage are depicted in Fig 7. *Scenedesmus quadricauda* strongly supported the branch points with bootstrap values 53–100% showing closest relationship with *Scenedesmus abundans* having minimum branch point distance of 0.0172. The bootstrap values range of phylogenetic tree of *Scenedesmus dimorphus* and *Chlorella* sp. were 18–71% and 33–100%, respectively. *Scenedesmus dimorphus* showed closest relationship with *Scenedesmus* sp. LG2VF16 and *Scenedesmus incrassatulus* with branch point distance of 0.0243 whereas *Chlorella* sp. was found to be phylogenetically nearest to *Chlorella emersonii* with branch point distance of 0.0347.

## Conclusion

On the basis of data obtained on growth parameters and lipid accumulation and profile, it is concluded that *Scenedesmus dimorphus* is a better potential biofuel feedstock compared to *S*. *quadricauda*. *Scenedesmus dimorphus* was also found to be suitable as biodiesel feedstock candidate on the basis of fatty acid profile analysis of total lipids. Two *Scenedesmus dimorphus* isolates viz. *Sd12* and *Sd1* out performed the rest with respect to important growth kinetic parameters. The lipid content of these isolates was found to be higher than most other species as reported in similar studies. These isolates can further be used in scale up experiments, improvements of culture and cultivation methods and even in genetic improvement studies. Molecular characterization of microalgae species under study was also performed using amplified 18S rRNA gene sequences. Amplified 18S rRNA gene fragments of the microalgae species were sequenced, annotated at the NCBI website and phylogenetic analysis was done. We have published eight 18S rRNA gene sequences of microalgae species in NCBI GenBank.

## Supporting Information

S1 Fig GC-MS chromatogram of microalgal lipid (FAME) of two microalgal species. (TIF)

S1 Table List of isolated microalgal isolates with different location of Himachal Pradesh, India. (DOCX)

S2 Table Fatty acid profile of total lipids of *Scenedesmus dimorphus* revealed via FAMEs detection by GC-MS. (DOCX)

S3 Table Fatty acid profile of total lipids of *Scenedesmus quadricauda* revealed via FAMEs detection by GC-MS. (DOCX)

S4 Table Fatty acid profile of total lipids of *Chlorella* sp. revealed via FAMEs detection by GC-MS. (DOCX)

S5 Table Results of sequence annotation of 18S rRNA gene fragment of microalgae species using BLAST tool at NCBI database. Similarity scores and accession numbers of ten most close sequences with query sequence of microalgae under study which were used for phylogenetic analysis are shown.
(DOCX)

## Abbreviations

OD: Optical density
LM: Light Microscope
SEM: Scanning Electron Microscope
FA: Fatty acid
FAMEs: Fatty acid methyl esters
Lp: Lipid productivity
Lc: Lipid content
Pdwt: Biomass productivity
SPSS: Statistical package for the social science
ANOVA: Analysis of variance
UFA: Unsaturated fatty acid
PUFAs: Polyunsaturated fatty acids
RT: Retention Time
MW: Molecular Weight
NIST: National Institute of Standard Technology
BG-11: Blue Green-11
BLAST: Basic Local Alignment Searching Tool
NCBI: National Centre for Biotechnology Information
GC-MS: Gas Chromatography Mass Spectroscopy
