# Supplementary figures and images for: Characterization and Screening of Native Scenedesmus sp. Isolates Suitable for Biofuel Feedstock
Source: PLoS One. 2016 May 19;11(5):e0155321. doi: 10.1371/journal.pone.0155321 (PMC4873191; doi:10.1371/journal.pone.0155321)

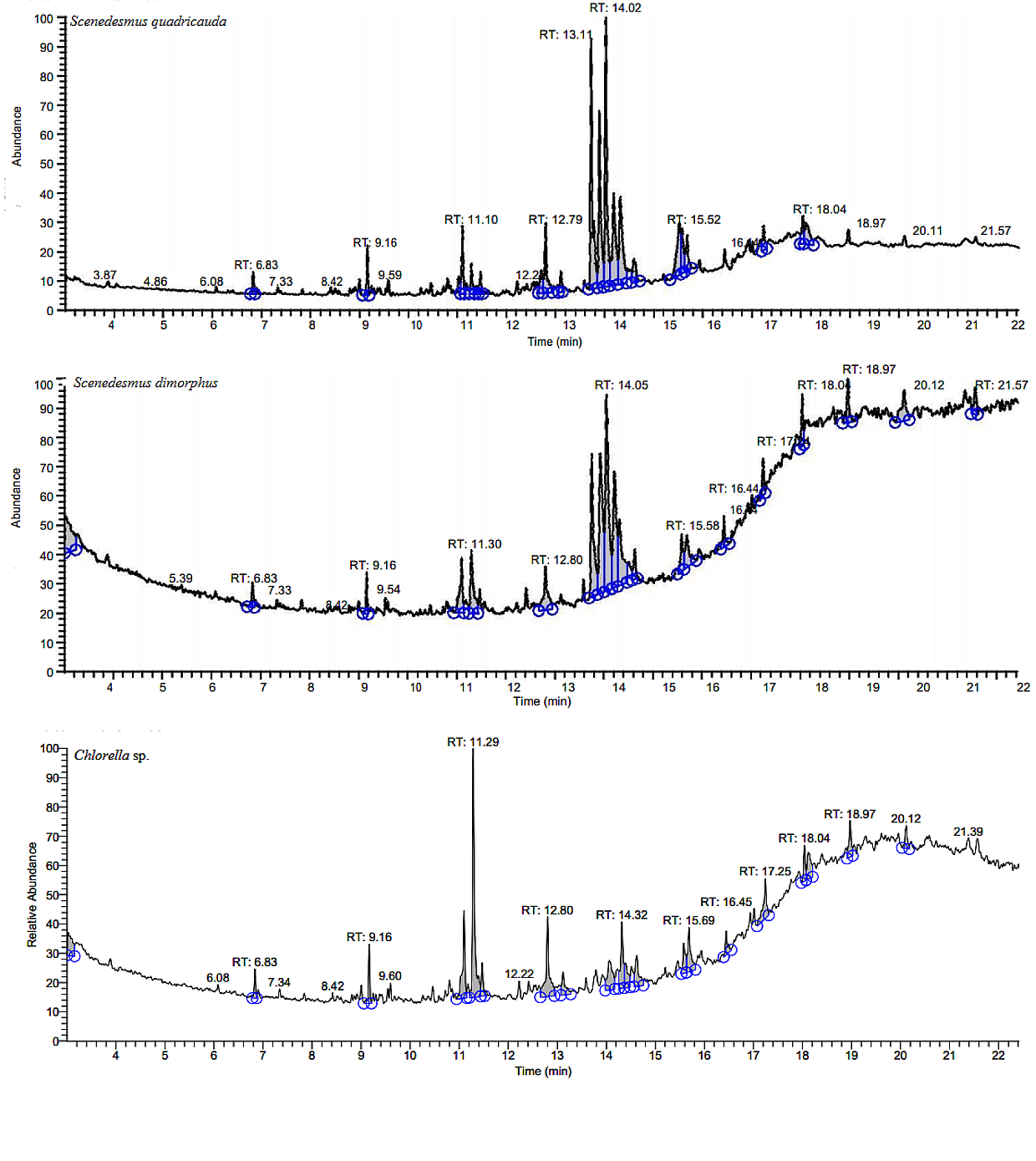

Supplement: S1 Fig — (TIF) [file pone.0155321.s001.tif]
