# Supplementary material for: Characterization and Screening of Native Scenedesmus sp. Isolates Suitable for Biofuel Feedstock
Source: PLoS One. 2016 May 19;11(5):e0155321. doi: 10.1371/journal.pone.0155321 (PMC4873191; doi:10.1371/journal.pone.0155321)
Supplement: S1 Table — (DOCX) [file pone.0155321.s002.docx]

**S1 Table** List of isolated microalgal isolates with different location of Himachal Pradesh, India

|  | | **Isolates** | | **Geographical coordinates** | **Location (site, city/region)** |
| --- | --- | --- | --- | --- | --- |
|  | *Sq1* | | *Sd1* | 31.343109, 76.761454 | Chamkhari pul, Bilaspur, Himachal Pradesh, India |
|  | *Sq2* | | *Sd2* | 31.343109, 76.761454 | Nalwar Jukhala, Bilaspur, Himachal Pradesh, India |
|  | *Sq3* | | *Sd3* | 31.343109, 76.761454 | Fish Breeding Farm (Deoli), Bilaspur, Himachal Pradesh, India |
|  | *Sq4* | | *Sd4* | 31.343109, 76.761454 | Seer Khad (Ghumarwin), Bilaspur, Himachal Pradesh, India |
|  | *Sq5* | | *Sd5* | 31.343109, 76.761454 | Rewal Tal (Dehra), Bilaspur, Himachal Pradesh, India |
|  | *Sq11* | | *Sd11* | 31.683365, 76.510345 | Kuhan Khad Rangus, Hamirpur, Himachal Pradesh, India |
|  | *Sq12* | | *Sd12* | 32.099952, 76.269236 | Deharian Kuna, Kangra, Himachal Pradesh, India |
|  | *Sq14* | | *Sd14* | 32.099952, 76.269236 | Baner Khad, Kangra, Himachal Pradesh, India |
|  | *Sq15* | | *Sd15* | 32.099952, 76.269236 | Kapur Sagar, Kangra, Himachal Pradesh, India |
|  | *Sq16* | | *Sd16* | 32.099952, 76.269236 | Fish Breeding Farm, Kangra, Himachal Pradesh, India |
|  | *Sq18* | | *Sd18* | 31.707832, 76.931183 | Ratti Khad, Mandi, Himachal Pradesh, India |
|  | *Sq19* | | *Sd19* | 31.707832, 76.931183 | Rewalshar Lake Mandi, Himachal Pradesh, India |
|  | *Sq20* | | *Sd20* | 30.904428, 77.096951 | Waknaghat, Solan Himachal Pradesh, India |
|  | *Sq21* | | *Sd21* | 30.904428, 77.096951 | Gambhar Khad, Solan Himachal Pradesh, India |

Note; *Sq*= *Scenedesmus quadricauda* and *Sd*= *Scenedesmus dimorphus*
