# Supplementary material for: Characterization and Screening of Native Scenedesmus sp. Isolates Suitable for Biofuel Feedstock
Source: PLoS One. 2016 May 19;11(5):e0155321. doi: 10.1371/journal.pone.0155321 (PMC4873191; doi:10.1371/journal.pone.0155321)
Supplement: S2 Table — (DOCX) [file pone.0155321.s003.docx]

**S2 Table** Fatty acid profile of total lipids of *Scenedesmus dimorphus* revealed via FAMEs detection by GC-MS

| Microalgae | RT | FAME | Carbon  Number | Molecular Mass | Cas No. | Relative content (%) |
| --- | --- | --- | --- | --- | --- | --- |
| *Scenedesmus dimorphus* | 3.02 | Butane, 2-methyl | C5:0 | 72.1 | 78-78-4 | 1.76 |
|  | 6.83 | Heptadecane, 9-hexyl | C23:0 | 324.6 | 55124-79-3 | 1.17 |
|  | 9.16 | Hexadecane | C16:2 | 226.4 | 544-76-3 | 1.51 |
|  | 11.10 | Tridecane, 2-methyl | C14:3 | 198.3 | NA | 4.03 |
|  | 11.30 | hexadecanoic acid (palmitic acid) | C16:0 | 256.4 | 57-10-3 | 10.14 |
|  | 12.80 | Phytol, acetate | C22:0 | 338.5 | NA | 4.42 |
|  | 13.75 | 3-Eicosyne | C20:1 | 278.5 | 61886-66-6 | 4.88 |
|  | 13.92 | Octadecatrienoic acid (linolenic acid) | C18:3 | 278.4 | 463-40-1 | 12.68 |
|  | 14.05 | Octadec-9-enoic acid (Oleic acid) | C18:1 | 282.4 | 112-80-1 | 16.12 |
|  | 14.21 | 9-Eicosyne | C20:0 | 278.5 | 71899-38-2 | 1.72 |
|  | 14.32 | 2-methyltetracosane | C25:0 | 352.6 | 1560-78-7 | 6.83 |
|  | 14.63 | Octadecadienoic acid (linoleic acid) | C18:2 | 280.4 | 60-33-3 | 10.11 |
|  | 15.58 | Tetradecamethyl Heptasiloxane | C14:0 | 505.0 | 19095-23-9 | 2.46 |
|  | 15.69 | Octadecane, 3-ethyl-5-(2-ethylbutyl) | C26:0 | 366.7 | 55282-12-7 | 3.49 |
|  | 16.44 | Octadecanoic acid (Stearic acid) | C18:0 | 284.4 | 57-11-4 | 6.10 |
|  | 17.24 | 9,12,15-Octadecatrienoic acid | C27:3 | 464.8 | 55521-22-7 | 1.55 |
|  | 18.04 | 1-Monolinoleoylglycerol trimethylsilyl ether | C27:2 | 498.8 | 54284-45-6 | 2.54 |
|  | 18.97 | Cyclodecasiloxane, eicosamethyl | C20:3 | 741.5 | 18772-36-6 | 2.81 |
|  | 20.12 | Heptasiloxane, hexadecamethyl | C16:3 | 533.1 | 541-01-5 | 3.80 |
|  | 21.57 | Hexasiloxane, 1,1,3,3,5,5,7,7,9,9,11,11-dodecamethyl | C12:1 | NA | 995-82-4 | 1.88 |

MUFA (Mono Unsaturated Fatty acid = 22.88%), PUFA (Poly Unsaturated Fatty acid = 39.03%), SFA (Saturated Fatty acid = 38.09%)
