# Supplementary material for: Characterization and Screening of Native Scenedesmus sp. Isolates Suitable for Biofuel Feedstock
Source: PLoS One. 2016 May 19;11(5):e0155321. doi: 10.1371/journal.pone.0155321 (PMC4873191; doi:10.1371/journal.pone.0155321)
Supplement: S3 Table — (DOCX) [file pone.0155321.s004.docx]

**S3 Table** Fatty acid profile of total lipids of *Scenedesmus quadricauda* revealed via FAMEs detection by GC-MS

| Microalgae | RT | FAME | Carbon  Number | Molecular Mass | Cas No. | Relative content (%) |  |
| --- | --- | --- | --- | --- | --- | --- | --- |
| *Scenedesmus*  *quadricauda* | 6.83 | Dodecane | C12:0 | 170.3 | 112-40-3 | 0.98 |  |
|  | 9.16 | Tetradecane | C14:0 | 198.3 | 629-59-4 | 2.05 |  |
|  | 11.10 | Octadecadicatrienoic acid (linolenic acid) | C18:3 | 278.4 | 463-40-1 | 6.21 |  |
|  | 11.28 | Phenol, 2,4-bis(1,1-dimethylethyl) | C14:1 | 206.3 | 96-76-4 | 1.77 |  |
|  | 11.46 | 9-Eicosyne | C20:0 | 278.5 | 1899-38-2 | 1.60 |  |
|  | 12.70 | Hexadecane | C16:2 | 226.4 | 544-76-3 | 1.81 |  |
|  | 12.79 | Octadecadienoic acid (linoleic acid) | C18:2 | 280.4 | 60-33-3 | 11.67 |  |
|  | 13.11 | Octadec-9-enoic acid (Oleic acid) | C18:1 | 282.4 | 112-80-1 | 15.60 |  |
|  | 13.72 | Phytol, acetate | C22:0 | 338.5 | NA | 1.03 |  |
|  | 13.89 | Eicosane, 2-methyl | C21:0 | 196.5 | 1560-84-5 | 4.69 |  |
|  | 14.02 | Hexadecanoic acid (palmitic acid) | C16:0 | 256.4 | 57-10-3 | 16.36 |  |
|  | 14.18 | 3,7,11-Trimethyl-2,4-dodecadiene | C15:0 | 208.3 | NA | 9.67 |  |
|  | 14.32 | Tetradecane, 2,6,10-trimethyl | C17:0 | 240.4 | 14905-56-7 | 9.12 |  |
|  | 14.59 | Heptadecane, 2,6,10,15-tetramethyl | C21:1 | 296.5 | 54833-48-6 | 2.13 |  |
|  | 15.52 | Methyl 8,11,14-heptadecatrienoate | NA | NA | NA | 3.45 |  |
|  | 15.58 | Propanoic acid | C27:1 | 430.6 | NA | 3.12 |  |
|  | 15.68 | 2-methylhexacosane | C27:0 | 380.4 | NA | 2.77 |  |
|  | 17.24 | Hexadecane, 2,6,11,15-tetramethyl | C20:1 | 282.5 | 504-44-9 | 1.19 |  |
|  | 18.04 | Octadecanoic acid (Stearic acid) | C18:0 | 284.4 | 57-11-4 | 1.70 |  |
|  | 18.11 | Hexa-t-butylselenatrisiletane | C24:0 | NA | 93194-15-1 | 3.07 |  |
|  | MUFA (Mono Unsaturated Fatty acid = 23.81%), PUFA (Poly Unsaturated Fatty acid = 19.69%), SFA (Saturated Fatty acid = 53.04%) | | | | | |  |
