# Supplementary material for: Characterization and Screening of Native Scenedesmus sp. Isolates Suitable for Biofuel Feedstock
Source: PLoS One. 2016 May 19;11(5):e0155321. doi: 10.1371/journal.pone.0155321 (PMC4873191; doi:10.1371/journal.pone.0155321)
Supplement: S4 Table — (DOCX) [file pone.0155321.s005.docx]

**S4 Table** Fatty acid profile of total lipids of *Chlorella* sp. revealed via FAMEs detection by GC-MS

| Microalgae | RT | FAME | Carbon  Number | Molecular Mass | Cas No. | Relative content (%) |
| --- | --- | --- | --- | --- | --- | --- |
| *Chlorella* sp. | 3.02 | Butane, 2-methyl | C5:0 | 72.1 | 78-78-4 | 4.15 |
|  | 6.83 | Octadecatrienoic acid (Linolenic acid ) | C18:3 | 278.4 | 463-40-1 | 1.72 |
|  | 9.16 | Pentadecane | C15:0 | 212.4 | 629-62-9 | 3.37 |
|  | 11.10 | Heptadecane, 2,6,10,15-tetramethyl | C21:1 | 296.5 | 54833-48-6 | 8.15 |
|  | 11.29 | hexadecanoic acid (Palmitic acid ) | C16:0 | 256.4 | 57-10-3 | 19.97 |
|  | 11.47 | Octadecane, 2-methyl | C19:0 | 296.5 | 54833-48-6 | 2.34 |
|  | 12.80 | Hexadecane, 2,6,11,15-tetramethyl | C20:2 | 282.5 | 504-44-9 | 9.85 |
|  | 13.12 | Heptadecane, 9-hexyl | C23:0 | 324.6 | 55124-79-3 | 3.03 |
|  | 14.06 | Phytol | C20:1 | 296.5 | 150-86-7 | 5.95 |
|  | 14.32 | Heneicosane, 11-(1-ethylpropyl) | C26:0 | 366.7 | 55282-11-6 | 6.27 |
|  | 14.39 | Ethyl iso-allocholate | C26:1 | 436.6 | NA | 3.37 |
|  | 14.62 | Octadec-9-enoic acid (Oleic acid) | C18:1 | 282.4 | 112-80-1 | 4.58 |
|  | 15.58 | Octadecadienoic acid (Linoleic acid ) | C18:2 | 280.4 | 60-33-3 | 2.91 |
|  | 15.69 | Octadecanoic acid (Stearic acid ) | C18:0 | 284.4 | 57-11-4 | 5.35 |
|  | 16.45 | Monolinoleoylglycerol trimethylsilyl ether | C27:0 | Not found | 54284-45-6 | 2.07 |
|  | 17.25 | Octasiloxane | C16:2 | Not found | 19095-24-0 | 4.94 |
|  | 18.04 | Heptasiloxane, hexadecamethyl | C16:3 | 533.1 | 541-01-5 | 2.42 |
|  | 18.12 | Dimethylpropanoilhydrazono) dichloro bis-[2-(diethylamino)-ethoxy]fluorene | C28:0 | 577.5 | NA | 4.03 |
|  | 18.97 | Cyclononasiloxane, octadecamethyl | NA | 667.3 | 556-71-8 | 3.32 |
|  | 20.12 | Cyclodecasiloxane, eicosamethyl | C20:3 | Not found | 18772-36-6 | 2.20 |

MUFA (Mono Unsaturated Fatty acid = 48.24%), PUFA (Poly Unsaturated Fatty acid = 22.05%), SFA (Saturated Fatty acid = 24.04%)
