# Supplementary material for: Characterization and Screening of Native Scenedesmus sp. Isolates Suitable for Biofuel Feedstock
Source: PLoS One. 2016 May 19;11(5):e0155321. doi: 10.1371/journal.pone.0155321 (PMC4873191; doi:10.1371/journal.pone.0155321)
Supplement: S5 Table — Similarity scores and accession numbers of ten most close sequences with query sequence of microalgae under study which were used for phylogenetic analysis are shown. (DOCX) [file pone.0155321.s006.docx]

**S5 Table** Results of sequence annotation of 18S rRNA gene fragment of microalgae species using BLAST tool at NCBI database. Similarity scores and accession numbers of ten most close sequences with query sequence of microalgae under study which were used for phylogenetic analysis are shown.

| *Scenedesmus quadricauda F4* | MAXScore | Query Cover | Max Ident |
| --- | --- | --- | --- |
| *Scenedesmus species KMMCC 1533* | 1273 | 98% | 98% |
| *Scenedesmus armatus var. subalternans* | 1273 | 98% | 98% |
| *Scenedesmus species KMMCC 1242* | 1271 | 98% | 98% |
| *S. abundans* (X73995.1) | 1271 | 98% | 98% |
| *Scenedesmus species KMMCC 1534* | 1260 | 98% | 98% |
| *Scenedesmus species KMMCC 1211* | 1260 | 98% | 98% |
| *Scenedesmus species KMMCC 178* | 1260 | 98% | 98% |
| *Scenedesmus species KMMCC 406* | 1256 | 98% | 98% |
| *Scenedesmus species KMMCC 872* | 1251 | 98% | 97% |
| *Scenedesmus dimorphus F4* | | | |
| *Scenedesmus bajacalifornicus strain ZA1-7* | 1290 | 96% | 99% |
| *Scenedesmus bajacalifornicus strain ZA1-4* | 1290 | 96% | 99% |
| *Scenedesmus bajacalifornicus strain BCP-MX15VF11* | 1290 | 96% | 99% |
| *Scenedesmus deserticola isolate BCP-HAF2-VF10* | 1290 | 96% | 99% |
| *Scenedesmus deserticola isolate BCP-YPG Char* | 1290 | 96% | 99% |
| *Scenedesmus deserticola isolate BCP-EM2-VF3* | 1290 | 96% | 99% |
| *Scenedesmus bajacalifornicus isolate BCP-LG2-VF34* | 1290 | 96% | 99% |
| *Scenedesmus species* LG2VF16 | 1290 | 96% | 99% |
| *Scenedesmus acuminatus* | 1290 | 96% | 99% |
| *Scenedesmus bajacalifornicus* strain ZA1-5 | 1288 | 95% | 99% |
| *Chlorella* sp*.* | | | |
| *Graesiella emersonii* | 1101 | 98% | 94% |
| *Chlorella emersonii* | 1101 | 98% | 94% |
| *Chlorella emersonii* | 1101 | 98% | 94% |
| *Graesiella emersonii* | 1050 | 94% | 94% |
| *Chlorella sorokiniana* | 1013 | 98% | 92% |
| *Chlorella sorokiniana* | 1013 | 98% | 92% |
| *Chlorella species* | 1013 | 98% | 92% |
| *Chlorella pulchelloides strain EN 2003/25* | 1013 | 98% | 92% |
| *Chlorella species SAG 222-2a* | 1013 | 98% | 92% |
